# Nanoneedles of Mixed Transition Metal Phosphides as Bifunctional Catalysts for Electrocatalytic Water Splitting in Alkaline Media

**DOI:** 10.3390/nano13040683

**Published:** 2023-02-09

**Authors:** Davide Salvò, Dario Mosconi, Alevtina Neyman, Maya Bar-Sadan, Laura Calvillo, Gaetano Granozzi, Mattia Cattelan, Stefano Agnoli

**Affiliations:** 1 Department of Chemical Sciences, University of Padova, Via Marzolo 1, 35131 Padova, Italy; 2 Avanzare Innovación Tecnológica S.L., Av. Lentiscares, 4-6, 26370 Navarrete, Spain; 3 Organometallic Molecular Materials (MATMO), Departamento de Química-Centro de Investigación en Síntesis Química (CISQ), Universidad de La Rioja, Madre de Dios, 53, 26006 Logroño, Spain; 4 Department of Chemistry, Ben Gurion University, Beer Sheva 84105, Israel

**Keywords:** water splitting, HER, OER, phosphide

## Abstract

In this work, mixed Ni/Co and Ni/Fe metal phosphides with different metal ratios were synthesized through the phosphidization of high-surface-area hydroxides grown hydrothermally on carbon cloth. The materials were characterized by means of X-ray photoemission spectroscopy, X-ray diffraction, energy dispersive X-ray analysis, and electron microscopies. The electrocatalytic performance in the electrochemical water splitting was tested in alkaline media. With the aim of determining the chemical stability of the mixed phosphides and the possible changes undergone under catalytic conditions, the materials were characterized before and after the electrochemical tests. The best performances in the hydrogen evolution reaction were achieved when synergic interactions are established among the metal centers, as suggested by the outstanding performances (50 mV to achieve 10 mA/cm^2^) of materials containing the highest amount of ternary compounds, i.e., NiCoP and NiFeP. The best performances in the oxygen evolution reaction were reached by the Ni-Fe materials. Under these conditions, it was demonstrated that a strong oxidation of the surface and the dissolution of the phosphide/phosphate component takes place, with the consequent formation of the corresponding metal oxides and hydroxides.

## 1. Introduction

Electrochemical water splitting is a key technology to cost-effectively produce hydrogen, which is regarded as the most promising energy vector for the replacement fossil fuels and the establishment of a new sustainable energy infrastructure. Currently, state-of-the-art electrocatalysts for water splitting are mainly based on platinum group metals (PGMs), such as Pt for the hydrogen evolution reaction (HER) and RuO_2_ or IrO_2_ for the oxygen evolution reaction (OER); however, their practical use in the market is hindered by their limited availability and high cost [1,2]. In recent years, great efforts have been made to develop highly efficient and sustainable non-PGM catalysts for the HER or OER [3,4]. These electrocatalysts include various transition metals combined with oxygen, nitrogen, boron, sulfur, and phosphorus [2,5,6,7,8].

Here, we focus on a series of mixed-transition metal (M) phosphides (M=Ni, Co, Fe) that are well. known for their chemical activity in electrochemical water splitting [9,10,11,12,13,14]. In particular, Ni and Co, are good candidates as electrocatalysts due to their thermal stability and good electrical properties [15,16]. To improve their electro-catalytic activity, tuning the chemical environment, structure, and morphology is critical [17]. In this work, we changed their chemical environment by preparing a series of mixed compounds (Ni/Co and Ni/Fe) to understand their reciprocal interaction and synergic effects. The interaction of different metals in close contact is an approach used to overcome the OER slow kinetics for water electrolysis and metal–air batteries [18,19,20,21,22]; this approach is also the founding principle of the chemical activity of high-entropy materials [23]. The combination of Ni and Co has been deeply investigated for HER, where the synergy among the catalytic properties of Ni, which lowers the HER over-potential, and Co, which increases the hydrogen adsorption, is realized and allows to obtain a larger value of exchange current density [24]. For OER, the Ni-Co system has unique redox characteristics in alkaline medium; for this material, water oxidation reactions usually involve proton-coupled electron transfer processes, i.e., the conversion of Co^3+^/Ni^3+^−OH to Co^4+^/Ni^4+^−O, prior to molecular oxygen evolution [25]. Fe is one of the most effective chemical additive to Ni catalysts, especially for promoting the OER. In fact, Fe^3+^ ions can increase electron conductivity and the reaction kinetics since these species have an optimal bond energetics for the adsorption of OER intermediates [26].

The phosphidization of the transition metals has been proven as an excellent way to improve their catalytic activity. M and P in M_x_P_y_ possess a partial positive charge (δ^+^) and a partial negative charge (δ^−^), respectively [9,10,11,12,13]. The bond formed between the metal and P can be ionic, covalent, or metallic depending on the stoichiometry of the compound and the nature of the atoms involved. P atoms in metal phosphides with more electronegativity can withdraw electrons from metal atoms, which can trap positively charged protons. A relatively strong M-P bond leads to phosphides with excellent mechanical stability and good resistance to oxidation and chemical attack [27]. Indeed, phosphides have good activity toward both the HER and the OER and exhibit corrosion stability in alkaline electrolytes. In the case of the OER, the good electrocatalytic performances are related to the formation of heterostructures, where metal oxo/hydroxo species form on the surface of the catalyst due to the surface oxidation of metal phosphides [28]. Furthermore, phosphides have much better conductivity properties than oxo-hydroxide [29,30].

Among the most common strategies for the synthesis of metal phosphides are solid-state synthesis at high temperature [31], reaction of trioctylphosphine (TOP) with organometallic complexes [32], and phosphidization of oxides or hydroxides [33].

The solid-state synthesis allows to obtain high-purity bulk materials, but it requires high temperatures and long reaction times. An example is the synthesis of iron phosphide, where a stoichiometric quantity of metallic iron and red phosphorus is placed in a sealed silica tube at 900 °C for eight days [34]. The synthesis by TOP can be performed with many transition metals and takes place at temperatures around 300 °C for a duration of a few hours [33]. This technique allows to prepare a variety of morphologies but generally exploits expensive precursors, and it is difficult to scale up.

The phosphidization of oxides or hydroxides can be achieved by heat treatment with phosphine (PH_3_ a highly toxic gas) or by the reduction of phosphates with hydrogen. We decided to use the latter strategy, as developed by Huang et al. [35], who grew a mixed phosphide of Ni and Fe on a carbon cloth (CC) using as template spherical structures composed by layered double hydroxides (LDHs) whose morphology was maintained even after the phosphidization treatment. We started from needle-like metal hydroxides containing Ni/Co and Ni/Fe in different ratios, which were grown by a hydrothermal route directly on CC, so that the final materials were characterized by a very open structure with a large amount of easily accessible active sites for electrocatalysis.

The physicochemical characterization of the resulting materials was performed by X-ray diffraction (XRD), X-ray photoemission spectroscopy (XPS), scanning and transmission electron microscopy (SEM, TEM), and energy dispersive X-ray (EDX) analysis. Electrocatalytic tests for the HER and OER were performed in semi-cells in alkaline media. The results prove that the formation of phosphide multi-metal solid solutions improves the performances in both reactions.

## 2. Materials and Methods

### 2.1. Materials Synthesis

Phosphides-based materials were grown on an additives-free carbon cloth (CC, Zoltektm PX30 Fabric, Bridgeton, MO, USA) using a two-step synthesis process. First, transition metal oxides were grown by hydrothermal synthesis. Second, the oxides were phosphidized in a tubular oven. To ensure a good adhesion of the oxides, the CC was subjected to a thermal treatment to modify the carbon surface from hydrophobic to hydrophilic [36]. CCs were annealed in air at 500 °C for 2 h. From the XPS measurements, which are not reported here, the oxygen content passed from 2.5% to 5.1% after the treatment.

The transition-metal oxides were grown by adding urea (NH_2_CONH_2_) (2.06 mmol) and water (13.8 mL) to the corresponding metal chlorides aqueous solution (1.03 mmol, Merck & Co Inc, Rahway, NJ, USA) containing the CC [37,38]. The synthesis was performed in a hydrothermal bomb at 120 °C for 4–8 h, and then, the sample was left to cool down naturally. The CC was washed with water and ethanol and dried under nitrogen atmosphere. The hydrothermal synthesis produced a complex mixture of oxides, hydroxides, and oxo-hydroxides. To facilitate the reading of the manuscript, we refer to them generally as oxides.

The phosphidization was carried out through the pyrolysis of sodium hypophosphite (NaH_2_PO_2_). The decomposition reaction begins around 100 °C and ends at 250 °C:

2 NaH_2_PO_2_ → PH_3_ + Na_2_HPO_4_

generating in situ PH_3_, which is the phosphidizing agent.

The transition from oxide to phosphide was chosen to increase the conductivity of the material; indeed, all the pure phosphide phases that we observed in our conditions, i.e., CoP, Ni_2_P, FeP, and Fe_2_P, have relatively small bandgaps of 1.71 eV, ≈1 eV, and 0–0.8 eV, respectively [29,30].

The metal-oxide-decorated CCs were placed inside an alumina boat and heated up to 300 °C under inert atmosphere for 2 h using a heating ramp of 3 °C/min. The phosphorus precursor (110 mg) was placed at a distance of about 7 cm from the center of the oven at a temperature of about 230–250 °C. To flush out any residual oxygen or water vapors, the system was purged with a flow of nitrogen at 1000 standard cubic centimeter per minute (SCCM) for ten minutes. Nitrogen played the role of carrier gas by transporting the PH_3_ to the CC at 70 SCCM.

Phosphide powder samples were synthesized by using the same procedure (without CC) and were used as XRD references.

### 2.2. Materials Characterization

XPS spectra were acquired in an ultra-high-vacuum (UHV) chamber with a working pressure below 5 × 10^−8^ mbar, using the Mg Kα line (hν = 1253.6 eV) and pass energy of 50 and 20 eV for survey and high-resolution spectra, respectively. The deconvolution into single chemical-shift components was carried out using the KolXPD software (Kolibri.net, Žďár nad Sázavou, Czech Republic) and Voigt functions.

SEM images were acquired using a Zeiss Supra VP35 (Zeiss, Oberkochen, Germany) with an acceleration voltage of 5 kV, using the signal coming from secondary electrons measured both by a conventional detector (Everhart-Thorley) and by an in-lens detector. EDX spectra were collected by the same instrument using higher acceleration voltage, depending on the metal present in the sample, between 14 kV and 18 kV.

XRD patterns were obtained by means of a Panalytical Aeris Research (Spectris, London, UK) instrument, using the Cu Kα radiation (λ = 0.15406 nm) generated at 30 kV and with a current of 15 mA. For instrumental reasons, the XRD spectra were acquired on powders prepared with the same synthetic methodology of the materials supported on CC (see Figure S1 in the Supplementary Materials). The powders were prepared starting from the solution produced by hydrothermal synthesis, filtered, and washed first with water and then with ethanol, which eventually was removed by a rotavapor. The phosphidization reaction of powders was carried out in the same way as for the CC-supported samples.

High-resolution transmission electron microscopy (HRTEM) imaging was carried out using a JEOL JEM-2100F analytical TEM (JEOL, Akishima, Tokyo, Japan) operating at 200 keV equipped with GATAN 894 US900 camera.

The electrochemical characterizations of all samples were performed in a four-electrode electrochemical cell. The reference electrode used was Ag/AgCl (3M KCl, 1.02 V vs. the reversible hydrogen electrode (RHE) at pH 14). Graphite plates were used as the counter electrodes. Two counter electrodes were used since each CC exposes two active faces, so the use of this geometry helped the current flows inside the cell. 1 M KOH solution (pH = 14) prepared with Milli-Q water was used as electrolyte. Before carrying out the HER measurements, N_2_ was bubbled to eliminate the air from the solution, and during the experiments, an atmosphere of N_2_ was maintained. The linear sweep voltammetry (LSV) measurements were carried out without agitation. The working electrode consisted of the phosphides-modified CC (1 cm × 1.5 cm) with the top 0.5 cm masked with Teflon-coated glass fiber to limit the deposited area. All the current densities presented in this work were normalized by the geometric area of the electrode (2 cm^2^) and corrected by the ohmic drop in the solution. The ohmic drop was determined by electrochemical impedance spectroscopy (EIS) measurements. XPS was carried out on electrochemically treated samples to understand the surface chemical changes. The electrochemical ageing was performed by sweeping 2000 cycles from 0.1 to −0.2 V for HER and from 0.9 to 1.7 V for OER, and XPS measurements were acquired immediately after the EC treatments to lower the air exposure/contamination as much as possible.

## 3. Results and Discussion

### 3.1. Physico-Chemical Characterization of Ni/Co Mixed Phosphides

The morphology of the oxides grown on CC was studied by SEM, showing needle-like features that were maintained also after the phosphidization treatment and equivalent heat treatment at 500 °C for 48 h in inert atmosphere (see Figure S2 for a complete set of data regarding the cobalt-based materials).

**Figure 1:**
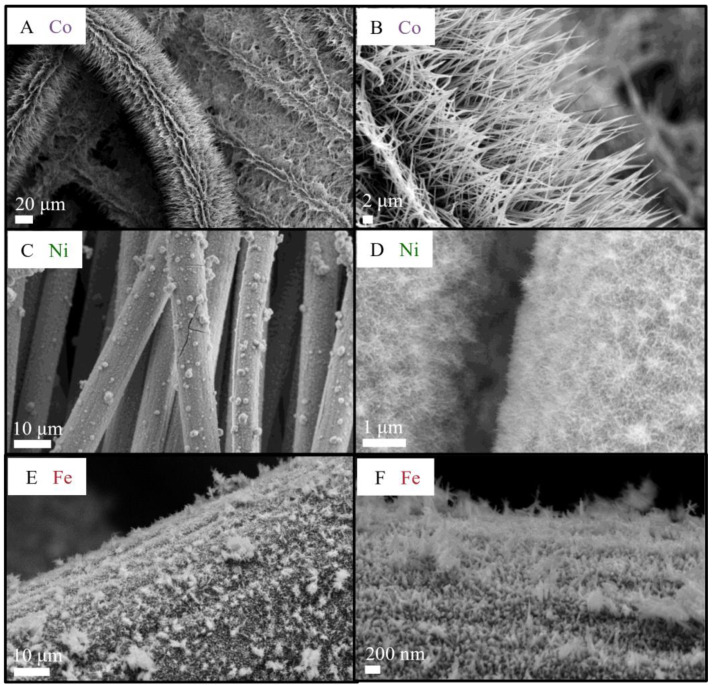
SEM images of Co, Ni, and Fe phosphides on CC at different magnifications. (**A**,**B**) CoP_x_, (**C**,**D**) NiP_x_, and (**E**,**F**) FeP_x_.

SEM images in Figure 1 show the differences between Ni and Co pure phosphides on CC. Both have a needle-like morphology; the CoP_x_ needles have a length of about 15 μm, an aspect ratio (i.e., length/width) larger than 10, and start from the CC fibers. The NiP_x_ needles, on the other hand, are significantly shorter, about 250 nm, and sometimes form spheres with a 2–3 μm diameter. They homogeneously cover the CC substrate.

Both CoP_x_ and NiP_x_ samples are excellent candidates for electrocatalysis since they have an open but highly connected hierarchical structure with a high surface area with easily accessible active sites for electrochemical reactions.

**Figure 2:**
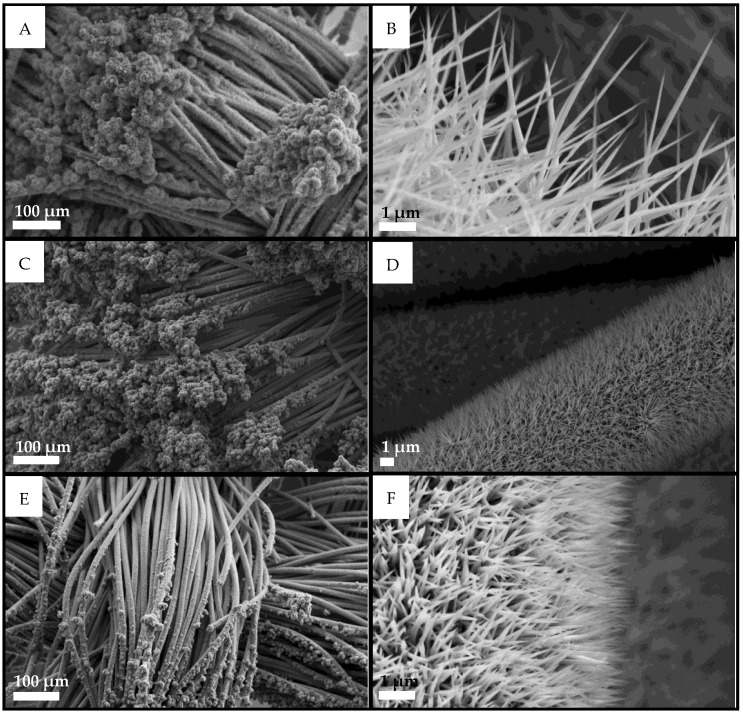
SEM images of mixed Ni and Co phosphides on CC at different magnifications. (**A**,**B**) Ni_0.25_Co_0.75_P_x_, (**C**,**D**) Ni_0.50_Co_0.50_P_x_, and (**E**,**F**) Ni_0.75_Co_0.25_P_x_.

Mixed phosphides were synthesized with atomic Ni:Co ratios of 25:75, 50:50, and 75:25. The morphology of Ni/Co mixed compounds (see Figure 2) is linked to the ratio between the relative quantities of the two metals: the needles become shorter as the content of Co is reduced and that of Ni increased, and their average size spans from 6 μm for Ni_0.25_Co_0.75_P_x_ down to 1–2 μm for the other two mixed phosphides. The needle features are visible on the CC body as well as for pure phosphides in Figure 1 and terminate with a macroscopic flower morphology at the end of the fiber.

EDX measurements confirmed a homogeneous distribution of the different metals and that the molar ratio between the metals in the precursors was maintained in the final materials. The atomic composition deduced form EDX is reported in Table 1 and was obtained after an average over different areas of the samples.

**Figure 3:**
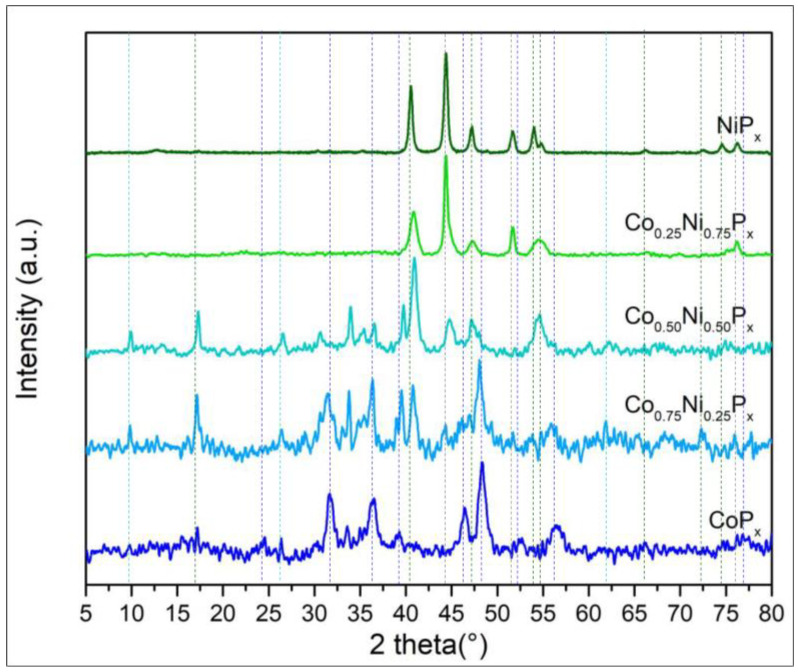
XRD patterns of Ni and Co phosphide alloys. The ratios between the two metals are those used for the preparation of the solutions used in hydrothermal bombs.

The XRD patterns in Figure 3 show three distinct phosphide phases: two related to the pure phases, namely Ni_2_P (green lines JCPDS No.03–0953) and CoP (blue lines JCPDS No.89–4862), and a phase given by the ternary compound of the two metals NiCoP (light blue lines JCPDS No.71–2336). For NiP_x_ that is pure or with a high Ni content, Ni metal features are visible at 44.1, 51.7, and 76.2° (grey lines JCPDS No. 01–1260). The metal Ni decreases drastically for the Co_0.75_Ni_0.25_P_x_, indicating the probable conversion of all Ni in phosphide compounds.

The ternary compound NiCoP has a hexagonal structure like that of Ni_2_P; therefore, it is difficult to identify exactly such a phase in the mixed-phosphide diffractogram. However, its presence is detectable also from a shift of the peak at 40.4° of Ni_2_P and the appearance of a peak at 26.5°.

**Figure 4:**
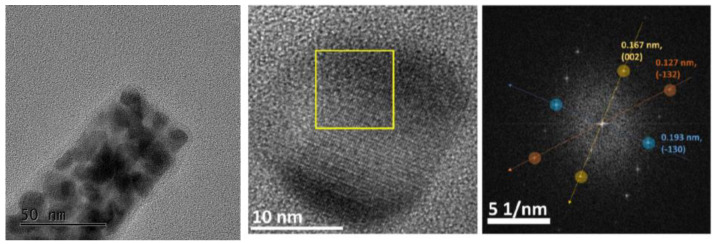
High-resolution TEM images of a Co_0.5_Ni_0.5_P_x_ needle. The Fourier transform acquired on the yellow square confirmed the Ni_2_P structure (space group P-62 m).

Comparing the spectra of pure NiP_x_ and CoP_x_ with the mixed one, we cannot exclude the co-presence of phase-pure Ni_2_P and CoP in the mixed compounds Co_0.75_Ni_0.25_P_x_ and Co_0.50_Ni_0.50_P_x_. Indeed, the presence of Ni_2_P in Co_0.50_Ni_0.50_P_x_ is confirmed by high-resolution TEM in Figure 4, where on a needle, 10 nm Ni_2_P particles are clearly identifiable by microdiffraction.

Interestingly, in the mixed phosphide Co_0.25_Ni_0.75_P_x_, a shift of the NiP_x_ peaks towards higher angles is visible, and the peaks associated with CoP_x_ are no longer observed, indicating the complete incorporation of Co into the NiCoP phase. This might be related to the higher EC activity in HER (see electrochemical tests paragraph).

TEM energy dispersive X-ray spectroscopy (EDS) was also performed to confirm the stoichiometry of the material. This technique showed a concentration of 21% at/at for both Co and Ni, confirming the metal ratio obtained by EDX (Table 1). However, the amount of P was only 18% at/at, and conversely, a high quantity of oxygen was detected: 40% at/at. This can be explained considering that TEM measurements were acquired some days after the materials preparation; therefore, the exposure to air could have produced some oxidation (see XPS below).

**Figure 5:**
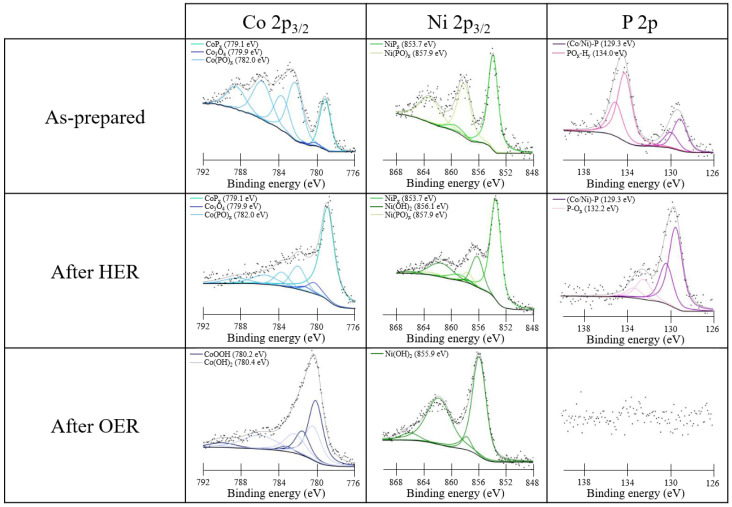
XPS spectra of the as-prepared Co_0.50_Ni_0.50_P_x_ sample on CC (**upper panels**) and after HER (**middle panels**) and OER (**bottom panels**) conditions.

XPS was used to identify the chemical species before and after the electrochemical measurements and on as-prepared CC. The sampling depth of XPS is only a few nanometers and therefore is indicative of the outer surface layer, whose composition is strongly dependent on the environment (exposure to air or to the electrolyte). The transition metal 2p_3/2_ photoemission lines along with P 2p were analyzed. Figure 5 shows the XPS analysis of the Co_0.50_Ni_0.50_P_x_ sample, while the results for Co_0.25_Ni_0.75_P_x_ and Co_0.75_Ni_0.25_P_x_ are reported in Figures S4 and S5, respectively. Similar trends were obtained for pure and mixed phosphides. To separate the photoemission spectra into chemically shifted components, we used the approach based on Gupta–Sen multiplets developed by Biesinger et al. [39].

In the spectra of the as-prepared samples, both phosphide and oxidized components are present. The presence of oxidized species is attributed to the oxidation of phosphides in contact with air. With regard to the Co 2p_3/2_ core level, three components are distinguished: CoP_x_ at a binding energy (BE) of 779.1 eV, Co(PO)_x_ at 782.0 eV, and a small amount of cobalt oxide Co_3_O_4_ at 779.9 eV [40]. In the case of the Ni 2p_3/2_ peak, only NiP_x_ at 853.7 eV and Ni(PO)_x_ at 857.9 eV are present, whereas hydroxide or oxide species are not detectable [41]. The P 2p photoemission line shows three components related to phosphide at 129.3 eV, P-O_x_ at 132.2 eV, and PO_x_-H_y_ at 134.0 eV [42].

In order to investigate the chemical stability of the samples under catalytic conditions, XPS measurements were acquired after electrochemical treatments under HER and OER conditions in alkaline conditions (middle and bottom panels in Figure 5). After the HER treatment, an increase of the phosphide component is observed, which becomes the major signal in the Co 2p, Ni 2p, and P 2p regions. The Co 2p and Ni 2p spectra also show components related to Co_3_O_4_, Co(PO)_x_, and Ni(OH)_2_, Ni(PO)_x_, respectively, but the oxidized components are less intense, suggesting that they were reduced or dissolved under HER conditions. Also in the P 2p region, a reduced amount of oxidized components is observed, and no PO_x_-H_y_ are detected.

TEM images of NiP_x_ needles before and after 2000 CV cycles from 0 to −0.35 V vs. RHE in HER regime in acid medium proved a strong modification of the sample morphology (see Figure S6). We expect a strong morphological modification also after ageing in alkaline media.

After the tests of OER activity, in all the cases, P is not detectable anymore, and Ni became prevalently hydroxides and Co a mixed hydroxide and oxo-hydroxide. The metal phosphides on the surface are completely oxidized to phosphates and dissolved, leaving only Co(OH)_2_, CoO(OH), and Ni(OH)_2_ [22,23]. Interestingly, for Co, the hydroxide phase is stabilized with respect to the oxo-hydroxide in the Co_0.25_Ni_0.75_P_x_ and prevalent for the other two mixed compounds. In the monometallic CoP_x_ sample (not reported here), the CoO(OH) and Co(OH)_2_ contents after OER are 61.6% and of 38.4% respectively, whereas in Co_0.25_Ni_0.75_P_x_, it is all converted in Co(OH)_2_ (see Figure S4). This can be linked to the formation of a ternary phase of phosphide that also changes the HER and OER performance (see the “Electrochemical tests” section).

### 3.2. Physico-Chemical Characterization of Ni/Fe Mixed Phosphides

Ni/Fe mixed phosphides were synthesized similarly to previous NiCoP_x_ samples [43]. In a similar synthesis, Huang et al. used sodium sulfate as the growth-promoting agent [35]. Since the interactions between the sulphate anion and the sodium cation with Ni under hydrothermal conditions are not known, we decided not to use any growth promoters and to use only urea as a precipitating agent [44].

For pure Ni and Fe oxides, it is possible to obtain a needle-like morphology even after 4 h of hydrothermal synthesis. The length of the Fe needles is slightly shorter than that of Ni and is about 50–100 nm. The Fe needles grow perpendicularly to the surface of the CC unlike those of Ni that tend to form spheres formed by radially oriented needles (see Figure 1).

After phosphidization, the composition was checked by EDX, and the results are summarized in Table 2. Additionally, in this case, the experimental values are in good agreement with the theoretical ones.

**Figure 6:**
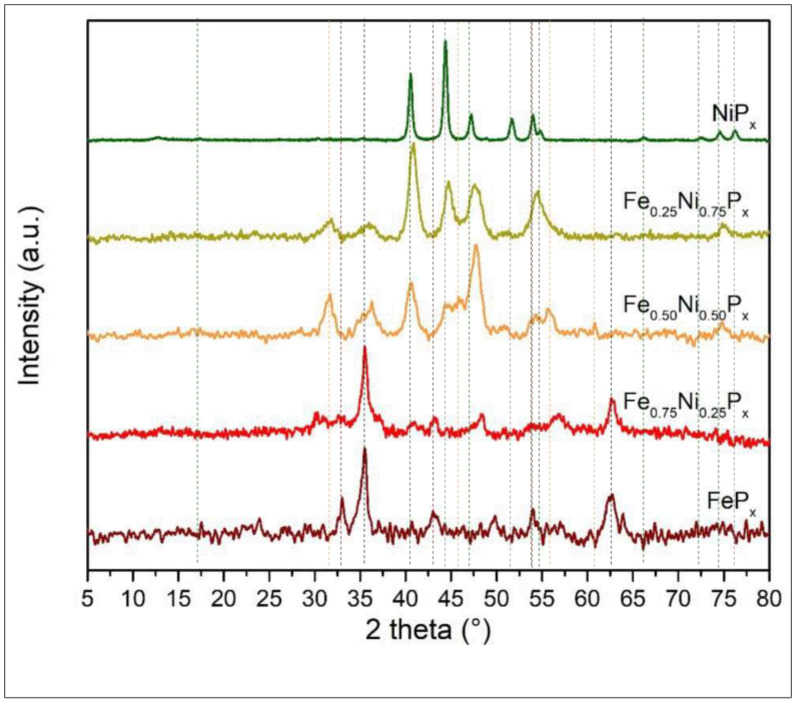
XRD pattern of Ni and Fe phosphide alloys. The ratios between the two metals are those used for the preparation of the solutions used in hydrothermal bombs.

Figure 6 reports the XRD patterns related to Ni and Fe phosphides. As for the NiCoP_x_ in Figure 3, we observed the distinctive peaks of NiP_2_ (green lines JCPDS No.03–0953) and metal Ni (grey lines JCPDS No. 01–1260); however, the quantity of metallic Ni reduces rapidly when mixed with Fe even at low concentration. In the FeP_x_ diffractogram (see also Figure S9), the Fe_2_P, FeP (dark red lines JCPDS No.88–1803, 89–2746), and Fe_3_O_4_ (black lines JCPDS No.03–0863) reflections are visible. The presence of Fe oxides for pure and mixed samples is proven by the intense magnetite signal at 35.5°. The tendency of Fe to be strongly oxidized is also confirmed by XPS.

The NiFeP (orange lines JCPDS No.36–1197) bimetallic phosphide solid solution shows a distinct peak at 31.7° associated with the (011) plane, which is already clearly discernible when the Ni and Fe are in a 3:1 ratio; the maximum intensity is reached when the metals are present with the same amount. Additionally, in this case, it is difficult to state if the mixed compound is made only by NiFeP or has some inclusions of FeP_x_/Fe_3_O_4_ and NiP_x_.

**Figure 7:**
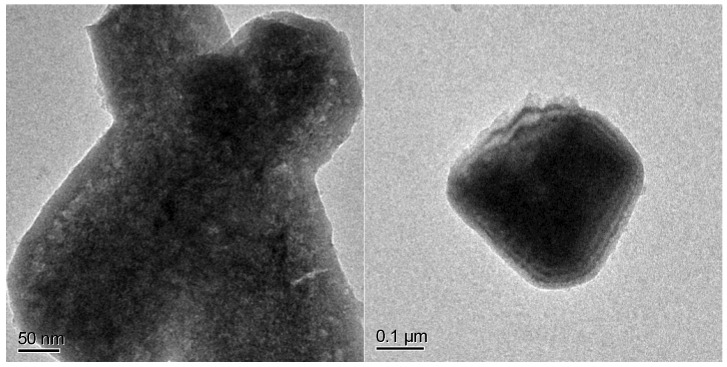
High-resolution TEM images of a needle of FeP_x_.

The presence of a considerable amount of magnetite in the as-prepared FeP_x_ is also visible by high resolution TEM. Figure 7 clearly shows a shell of different material, most probably an amorphous oxide, given the light featureless contrast, which is visible around a needle of FeP_x_.

**Figure 8:**
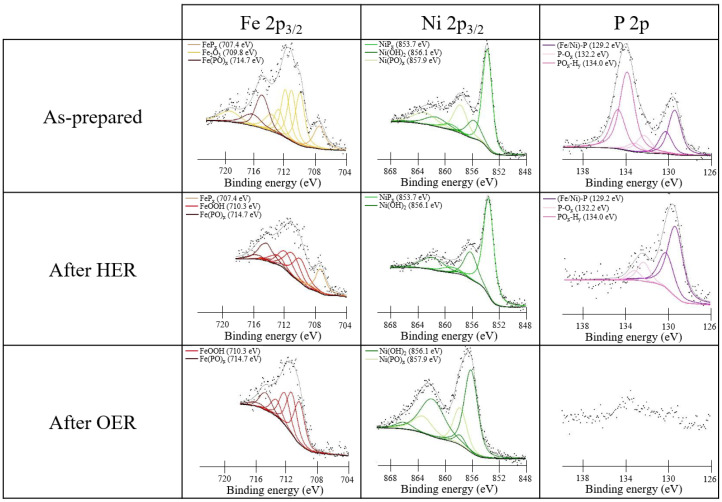
XPS data of the as-prepared Fe_0.50_Ni_0.50_P_x_ sample on the CC (**upper panels**) and after HER (**middle panels**) and OER (**bottom panels**) conditions.

Figure 8 reports the photoemission spectra of the Fe_0.50_Ni_0.50_P_x_ sample. Data for other combinations of the two metals are reported in Figures S7 and S8. To interpret such XPS data, we adopted the procedure based on Gupta–Sen multiplets developed by Biesinger. [39].

The Fe 2p_3/2_ BE region of the as-prepared Fe_0.50_Ni_0.50_P_x_ sample (Figure 8) shows at high BEs the presence of oxidized components in addition to those related to FeP_x_, i.e., ferric oxide and iron phosphate, with both due to the air exposure. According to the literature, the Fe 2p_3/2_ BE value of the peak assigned to Fe phosphide is similar for FeP and Fe_2_P (707.4 eV), and also, from our XRD measurements, it was found that both Fe_2_P and FeP were present (see Figure S9), so we cannot infer the exact stoichiometry of the Fe phosphide [45].

Regarding the Fe 2p_3/2_ region after HER in alkaline electrolyte, it is possible to note again three components, i.e., the FeP_x_, which remains fixed at 707.4 eV, the iron oxo-hydroxide FeO(OH) at 710.3 eV, and the iron phosphate at 714.7 eV [42]. The presence of oxo-hydroxides was already observed in the CC sample with Co and Ni phosphides (see Figure 5); therefore, we can confirm the tendency to form these phases in an alkaline environment at a reducing potential. After OER, the spectrum of Fe 2p_3/2_ shows mainly the FeO(OH) signal at 710.3 eV and some phosphate.

Additionally, in the case of the Ni 2p_3/2_ spectra, the observed components are very similar to those present in CoNiP_x_ (Figure 5). The only difference is the more limited oxidation of Ni probably due to the competitive oxidation of Fe.

The P 2p signal shows substantial similarities with that measured in Co_0.50_Ni_0.50_P_x_ (Figure 5). For the as-prepared sample, three different components are present: one related to Fe-Ni P_x_ at 129.1 eV and two oxidized components, i.e., the signal of the P-O_x_ at 132.2 eV and the one for PO_x_-H_y_ at 134.0 eV. These three components are also maintained after HER with a strong reduction of the P-O_x_ species. As in the case of the Co_0.50_Ni_0.50_P_x_ sample, after OER, the P 2p signal decreases severely, indicating only the presence of some phosphate residues in both the Fe 2p_3/2_ and Ni 2p_3/2_ spectra. This proves that by applying an oxidizing potential in an alkaline environment, most of the P is removed from the surface.

### 3.3. Electrochemical Tests

The electrochemical measurements were carried out in alkaline medium. The Co, Ni, and Fe phosphides showed excellent chemical resistance under HER conditions, as is visible by the XPS characterization above. In this paragraph, we discuss only measurements in 1 M KOH. HER measurements in acidic environment are reported in Figures S10 and S11.

Figure S12 reports the performance of the mixed Co and Ni phosphide compared to pure CoP_x_ and NiP_x_. The HER performance follows a clear trend as a function of the Ni content, and the highest activity is achieved with the Ni_0.75_Co_0.25_P_x_, i.e., the alloy with majority of Ni. It is interesting to see that pure NiP_x_ is less active than CoP_x_, proving that in the mixed phosphides the catalytic activity is not simply the sum of the single metal phosphide performance but is due to a synergic effect of the metals. Indeed, the XRD data (see Figure 3) suggest that the mixed phosphide Ni_0.75_Co_0.25_P_x_ presents the highest amount of NiCoP ternary phase. Tafel plots are reported in Figure S12 as well; their values range from 43 to 57 mV/dec, with the lowest value for Ni_0.75_Co_0.25_P_x_ and the highest one for NiP_x_. Values close to 40 mV/dec indicate that the rate determining step is the electrochemical hydrogen desorption according to the Heyrovsky process.

Figure S13 reports the LSV for the mixed Fe and Ni phosphides and for reference the data of the monometallic FeP_x_ and NiP_x_. In this case, there is not a clear trend of improvement with the quantity of Ni. The best overpotential to reach 10 mA/cm^2^ is obtained with Ni_0.50_Fe_0.50_P_x_. Nonetheless, these data also suggest that the electrochemical performances are not the simple combination of those of FeP_x_ and NiP_x_, which in this case are very similar. The most active combination of metals is that exhibiting the stronger signal related to the FeNiP ternary compound in the XRD pattern (see Figure 6), once again linking enhanced performances to the formation of the ternary phosphide, as for Ni/Co samples. Tafel plots are reported in Figure S13, and the values range from 62 to 75 mV/dec, which are therefore higher than those for the Ni/Co samples. However, values close to 40 mV/dec can still indicate that the rate determining step is the Heyrovsky process or the combination of the Heyrovsky desorption and the Volmer adsorption.

**Figure 9:**
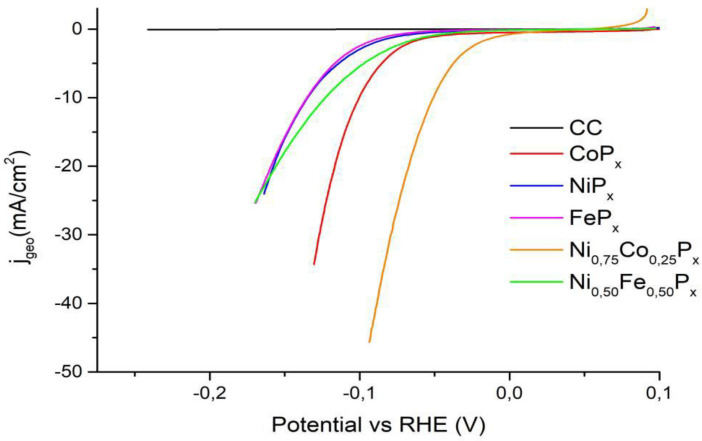
LSVs in 1 M KOH on the most performing mixed-phosphides samples. The HER activity of the pure Co, Ni, and Fe phosphides is included for comparison.

Figure 9 reports the LSVs for the most active mixed phosphides toward HER.

Table 3 summarizes the comparison of our results and the best phosphide-based catalysts reported in the literature. In general, the mixed phosphides show a higher activity than pure phosphides. In the case of Ni_0.75_Co_0.25_P_x_, a shift of 84 mV and 50 mV toward less negative values of the η_10_ is observed compared to NiP_x_ and CoP_x_, respectively (see Table 3). For Ni_0.50_Fe_0.50_P_x_, the improvement with respect to NiP_x_ and FeP_x_ is less evident, but a shift of around 12 mV toward less negative potentials is observed.

The values reported in the literature are comparable to those obtained in this work. It should be noted that the value reported for FeP_x_ (96 mV) [43] refers to a catalyst that had undergone a pre-treatment in acid to remove the surface phosphate and increase the electrochemical performance; therefore, it is not directly comparable to ours. The overpotential of 50 mV to reach 10 mA observed for Ni_0.75_Co_0.25_P_x_ places this material at the top of the activity among most performing materials in the literature.

In Figures S14 and S15, the OER measurements in alkaline media for all the samples with Co/Ni and Fe/Ni mixed phosphide are reported, respectively. Interestingly, the best-performing electrodes in alkaline HER are the worst mixed phosphides in OER. As discussed previously, the XPS analysis proves that after OER, the surface is covered by a majority of hydroxide and oxo-hydroxide, and the redox properties depend on the amount of ternary solid solution in the material.

The best-performing electrode for the Co-Ni system is the Ni_0.50_Co_0.50_P_x_, but it has very similar characteristics as the pure NiP_x_, CoP_x_, and Ni_0.25_Co_0.75_P_x_, and the OER performances seem scarcely dependent on the composition and just as unfavorable when the ternary compound is formed. Tafel plots, as reported in Figure S14, go from 63 mV/dec for NiP_x_ to 186 mV/dec for Ni_0.75_Co_0.25_P_x_, a d the high Tafel slopes values reflect the complex mechanism of OER for these materials. For pure Co compounds, the following mechanism has been proposed:

Co^II^ + 3OH^−^ ↔ Co^III^OOH + H_2_O + e^−^

Co^III^OOH + OH^−^ ↔ Co^IV^O(OH)_2_ + e^−^

Co^IV^O(OH)_2_ + 2OH^−^ ↔Co^IV^OO_2_ + 2H_2_O + 2 e^−^

Co^IV^OO_2_ + OH^−^ → Co^III^OOH + O_2_(g) + e^−^

with first three steps reversible and crucial for the reaction rate [50].

On the other hand, for the Ni-Fe mixed phosphides, the presence of Ni drastically increases the performance of pure FeP_x_, and the compositions with 25% and 75% of Ni have similar proprieties. Considering that FeP_x_ has the worst performance of this set, we link the lower overpotential of these two CCs to precipitation of NiP_x_. Interestingly, Ni/Fe LDH are one of the most studied as an efficient electrocatalyst for OER in alkaline media. The most active Ni/Fe LDH have Ni:Fe ratios ranging from 3 to 4 [51]; we confirm this result, with the most performing catalyst being the Ni_0.75_Co_0.25_P_x_ with Ni:Fe ratio of 3. Tafel plots in Figure S15 report a slope value ranging from 60mV/dec for NiP_x_ and 230 mV/dec for Ni_0.50_Fe_0.50_P_x_. NiP_x_ performances are very similar for the Ni/Co and Ni/Fe series; therefore, it can be concluded that they are not strongly influenced by the lower hydrothermal growth time for Ni/Fe series.

**Figure 10:**
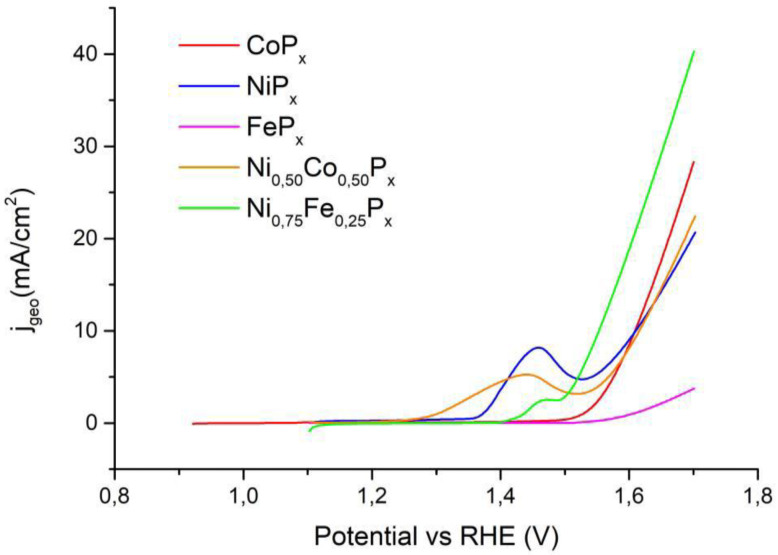
Measurements of electrochemical activity for OER in alkaline electrolyte for the best-performing mixed phosphide and pure Co, Ni, and Fe phosphides.

Figure 10 reports the OER measurements for the best-performing catalysts, while Table 4 summarizes the η_10_ values. In the case of FeP_x_, the current value of 10 mA/cm^2^ was not reached.

In the OER, the mixed Ni_0.50_Co_0.50_P_x_ material gives slightly worse results with respect to Ni and Co pure phosphides even if the difference is minimal. As for the Ni_0.50_Fe_0.50_P_x_ phosphide, it can be observed that the overpotential is significantly lower than that of NiP_x_ (58 mV), and it is clearly better compared to FeP_x_, which cannot reach the target current density. All our compounds perform better than similar materials reported in the literature data, as can be seen from the data in Table 3 and Table 4. This can be attributed to the high-surface-area needle-like electrodes obtained by the hydrothermal synthesis of the oxides.

## 4. Conclusions

In this work, three different transition metal phosphides have been proposed for the electrochemical splitting of water in alkaline medium.

Two mixed compounds were synthesized: Ni/Co and Ni/Fe phosphides with different metal ratios. The reason for synthesizing mixed phosphides was to establish possible synergistic effects between the two metals, which can potentially produce an increased catalytic activity.

CC was used as a substrate for the growth of the materials in order to maximize the active surfaces area of the resulting catalysts. The preparation procedure implied a first step for the preparation of hydroxides and oxo-hydroxides, which in a second stage underwent phosphidization. We demonstrated that the needle morphology of the precursor oxides on CC was maintained after phosphidization and that this process improved the electron-conducting properties, increasing the electrocatalytic activity of the materials.

This study systematically investigated how the relative quantities of the two metals influence the electrocatalytic properties of the phosphides in the electrochemical water splitting. For HER, the presence of ternary phosphide compounds, i.e., NiCoP and NiFeP, is crucial to increase the material performances. Indeed, NiCoP and NiFeP ternary phases are known as excellent catalysts due to the synergistic effects among the metals, whose exact nature is still highly debated [9,10,11,12,13,28]. In particular, the Ni_0.75_Co_0.25_P_x_ sample achieved an overpotential of only 50 mV to reach 10 mA/cm^2^, which is a value rarely matched in the literature in alkaline conditions.

In OER conditions, on the other hand, the establishment of precise links between chemical composition and activity is more difficult because the phosphides are converted to oxo-hydroxides during working conditions, whose precise structure is related to the phosphide precursors in a complex way. For Ni/Fe mixed compounds, we explain that within our selected combination of metal percentage, the most active material is the one with most NiP_x_ because of the poor performance of FeP_x_. For Co/NiP_x_, which are both good catalysts for OER, the performances are almost independent on the composition and very similar to the pure metal phosphides.

Finally, it should be highlighted that our synthesis protocol is simple, safe, and cost-effective, and it leads to catalysts with excellent activity. Therefore, with further durability studies, they can be considered for application in the development of real electrolyzers.

## Figures and Tables

**Table 1:** EDX stoichiometry results for Ni/Co mixed phosphides on CC.

Materials	Ni:Co Theoretical	Ni:Co Experimental	M:P Experimental
Ni_0.25_Co_0.75_P_x_/CC	25:75	26:74	55:45
Ni_0.5_Co_0.5_P_x_/CC	50:50	52:48	46:54
Ni_0.75_Co_0.25_P_x_/CC	75:25	73:27	45:55

**Table 2:** EDX stoichiometry results for Ni/Fe mixed phosphides.

Materials	Ni:Fe Theoretical	Ni:Fe Experimental	M:P Experimental
Ni_0.25_Fe_0.75_P_x_	25:75	23:77	46:54
Ni_0.5_Fe_0.5_P_x_	50:50	55:45	53:47
Ni_0.75_Fe_0.25_P_x_	75:25	78:22	54:46

**Table 3:** HER overpotentials to reach 10 mA/cm^2^ (η_10_) in 1 M KOH.

Materials	η_10_ (mV)	
	Our Data	Literature
CoP_x_	100	95 [46]
NiP_x_	134	115 [47]
FeP_x_	135	96 [43]
Ni_0.75_Co_0.25_P_x_	50	53 [48]
Ni_0.50_Fe_0.50_P_x_	123	161 [49]

**Table 4:** Potentials to be applied to the electrodes to reach 10 mA/cm^2^ (η_10_) in alkaline electrolyte in the OER.

Materials	η_10_ (mV)	
	Our data	Literature
CoP_x_	379	580 [47]
NiP_x_	380	400 [52]
FeP_x_	/	
Ni_0.50_Co_0.50_P_x_	385	660 [53]
Ni_0.75_Fe_0.25_P_x_	322	359 [35]

## Data Availability

The data that support the findings of this study are available from the corresponding authors upon reasonable request.

## Supplementary Materials

The following supporting information can be downloaded at: https://www.mdpi.com/article/10.3390/nano13040683/s1. The following supporting information can be downloaded at: Figure S1: XRD comparison CoOx on CC and CoOx powder; Figure S2: SEM images of CoOx on CC after different heat treatment; Figure S3: XRD spectra of Ni and Co oxides alloys; Figure S4: XPS data of Co0.25Ni0.75Px on CC; Figure S5: XPS data of Co0.75Ni0.25Px on CC; Figure S6: TEM picture of NiPx before and after acidic ageing; Figure S7: XPS data Fe0.25Ni0.75Px on CC; Figure S8: XPS data of Fe0.75Ni0.25Px on CC; Figure S9: XRD spectrum of FePx; Figure S10: HER EC in acid electrolyte for CC with Co and Ni phosphides; Figure S11: HER EC in acid electrolyte for CC with Fe and Ni phosphides; Figure S12: HER EC in alkaline electrolyte for CC with Co and Ni phosphides; Figure S13: HER EC in alkaline electrolyte for CC with Fe and Ni phosphides; Figure S14: OER EC in alkaline electrolyte for CC with Co and Ni phosphides; Figure S15: OER EC in alkaline electrolyte for CC with Fe and Ni phosphides.
