# Supplementary material for: Nanoneedles of Mixed Transition Metal Phosphides as Bifunctional Catalysts for Electrocatalytic Water Splitting in Alkaline Media
Source: Nanomaterials (Basel). 2023 Feb 9;13(4):683. doi: 10.3390/nano13040683 (PMC9963911; doi:10.3390/nano13040683)
Supplement: Supplementary file 1 [file nanomaterials-13-00683-s001.zip › nanomaterials-2152244-supplementary.pdf]

nanomaterials

MDPI

Supplementary Materials

Nanoneedles of Mixed Transition Metal Phosphides As Bifunctional Catalysts for Electrocatalytic Water Splitting in Alkaline Media

Davide Salvò [1,2,3], Dario Mosconi [1], Alevtina Neyman [4], Maya Bar-Sadan [4], Laura Calvillo [1], Gaetano Granozzi [1], Mattia Cattelan [1,*] and Stefano Agnoli [1,*]

[1] Department of Chemical Sciences, University of Padova, Via Marzolo 1, 35131 Padova, Italy
[2] Avanzare Innovación Tecnológica S.L., Av. Lentiscares 4-6, 26370 Navarrete, Spain
[3] Organometallic Molecular Materials (MATMO), Departamento de Química-Centro de Investigación en Síntesis Química (CISQ), Universidad de La Rioja, Madre de Dios 53, 26006 Logroño, Spain
[4] Department of Chemistry, Ben Gurion University, Beer Sheva 84105, Israel
* Correspondence: mattia.cattelan@unipd.it (M.C.); stefano.agnoli@unipd.it (S.A.); Tel.: +39-0498275845 (M.C.); +39-0498275167 (S.A.)

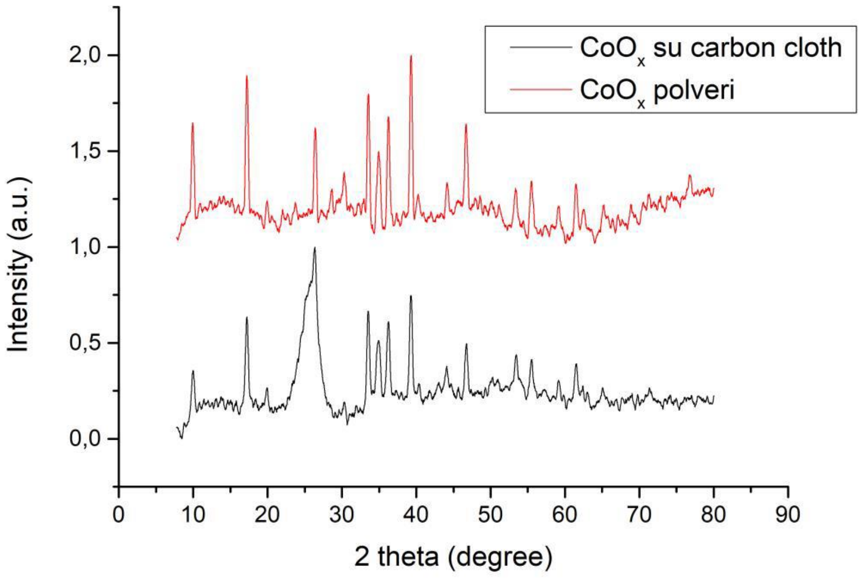

Figure S1. XRD patterns of CoO_x on CC (black) and CoO_x powder (red).

The two spectra are perfectly superimposable. The most visible difference is the peak at 26° in CoO_x on CC, which is related to the (002) crystallographic plane of graphite from CC, since it is made up of 99% carbon.

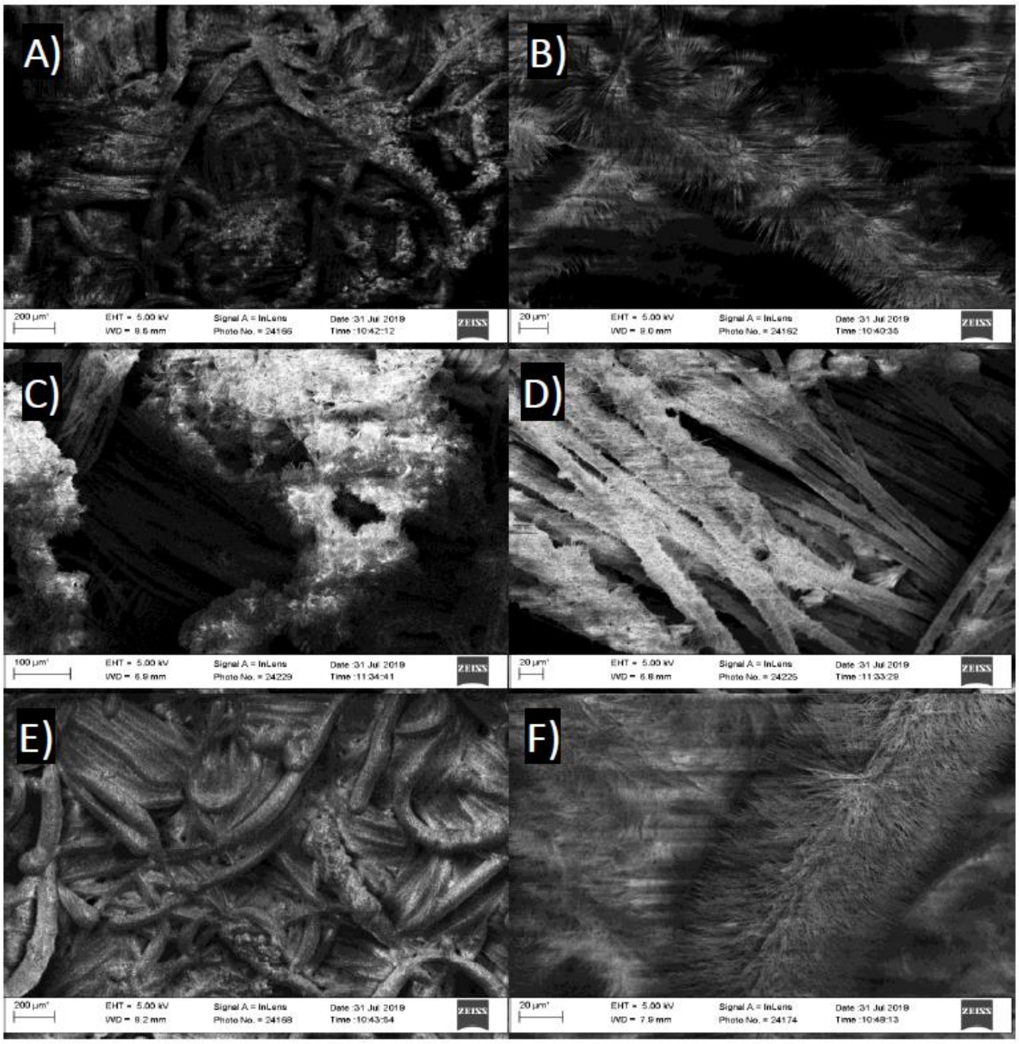

Figure S2. SEM images of CoO_x on CC after different heat treatments in inert atmosphere: (**A**,**B**) heat treatment at 500 °C for 2 h, (**C**,**D**) heat treatment at 500 °C for 6 h, and (**E**,**F**) heat treatment at 500 °C for 48 h.

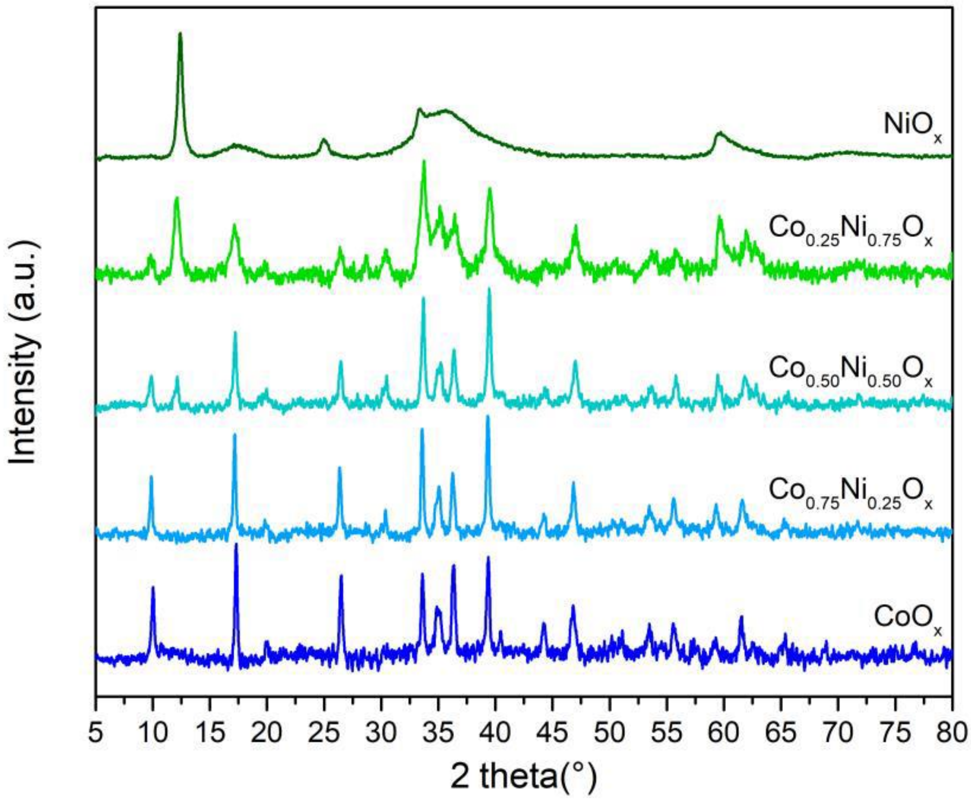

Figure S3. XRD patterns of pure and mixed Ni and Co oxides.

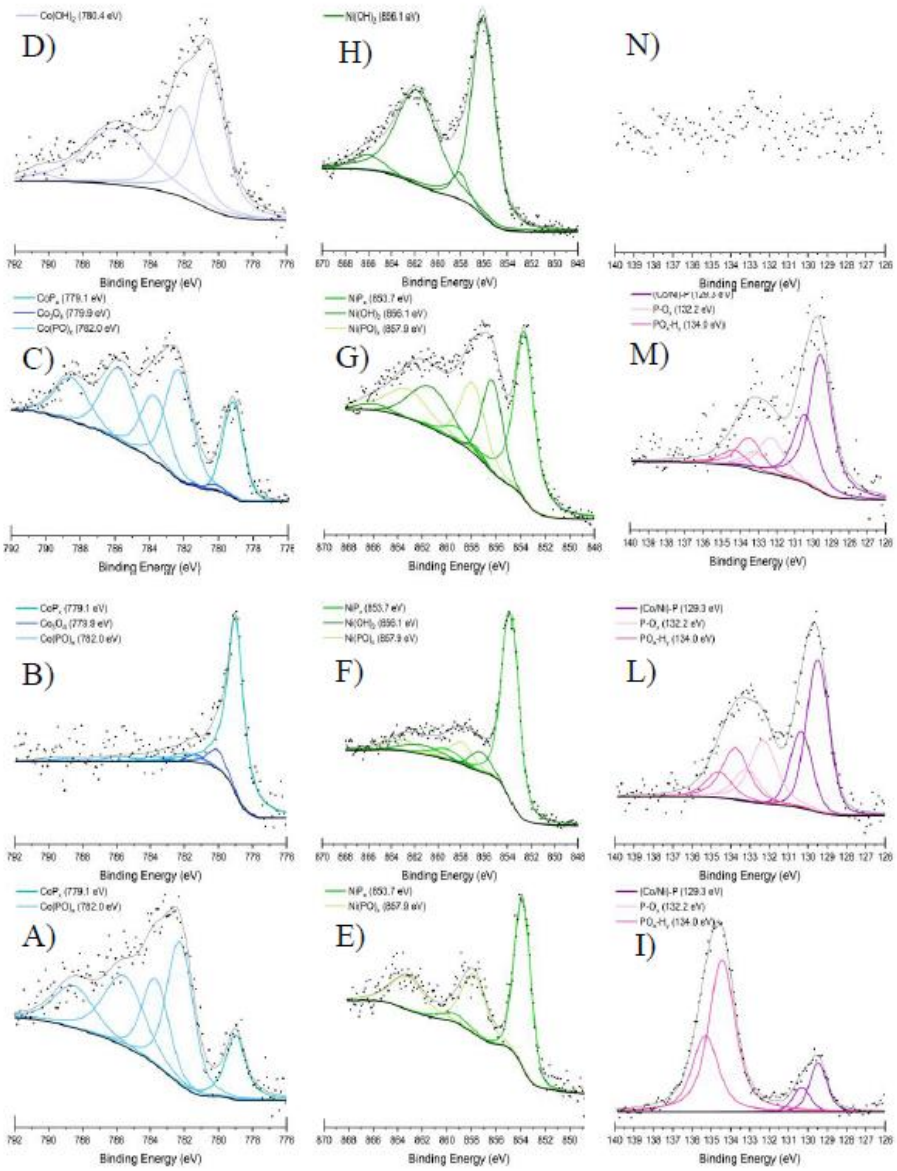

Figure S4. XPS data of $Co_{0.25}Ni_{0.75}P_x$ on CC: (**A**) Co 2p as-prepared, (**B**) Co 2p post-HER in acid solution, (**C**) Co 2p post-HER in alkaline solution, (**D**) Co 2p post-OER in alkaline solution, (**E**) Ni 2p as-prepared, (**F**) Ni 2p post-HER in acid solution, (**G**) Ni 2p post-HER in alkaline solution, (**H**) Ni 2p post-OER in alkaline solution, (**I**) P 2p as-prepared, (**L**) P 2p post-HER in acid solution, **M**) P 2p post -HER in alkaline solution, (**N**) P 2p post-OER in alkaline solution.

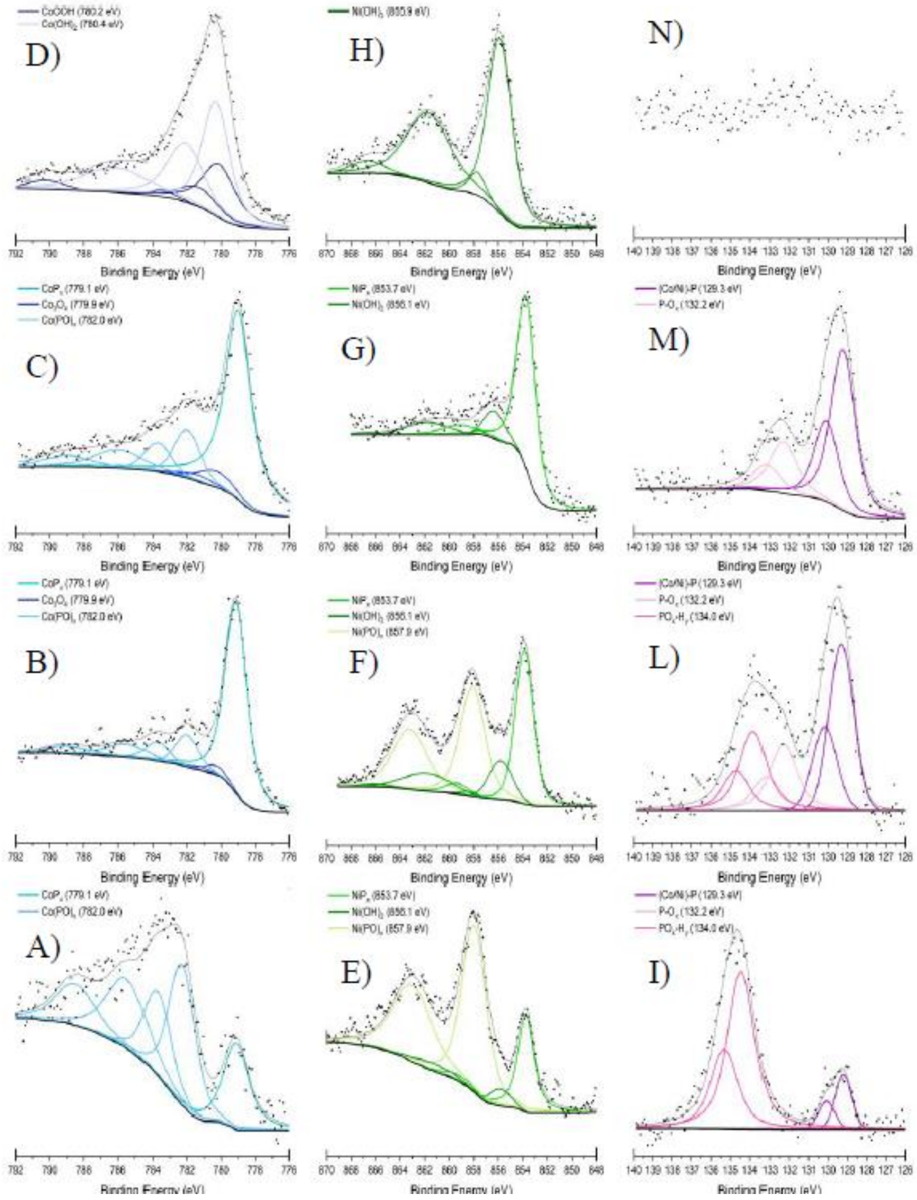

Figure S5. XPS data of $Co_{0.75}Ni_{0.25}P_x$ on CC: (**A**) Co 2p as-prepared, (**B**) Co 2p post-HER in acid solution, (**C**) Co 2p post-HER in alkaline solution, (**D**) Co 2p post-OER in alkaline solution, (**E**) Ni 2p as-prepared, (**F**) Ni 2p post-HER in acid solution, (**G**) Ni 2p post-HER in alkaline solution, (**H**) Ni 2p post-OER in alkaline solution, (**I**) P 2p as-prepared, (**L**) P 2p post-HER in acid solution, (**M**) P 2p post -HER in alkaline solution, (**N**) P 2p post-OER in alkaline solution.

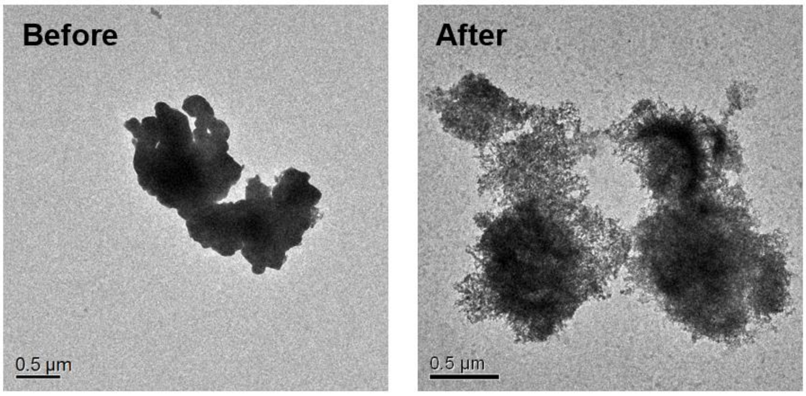

Figure S6. High-resolution TEM images of NiP_x needles before and after the electrochemical HER cycling treatment in acid.

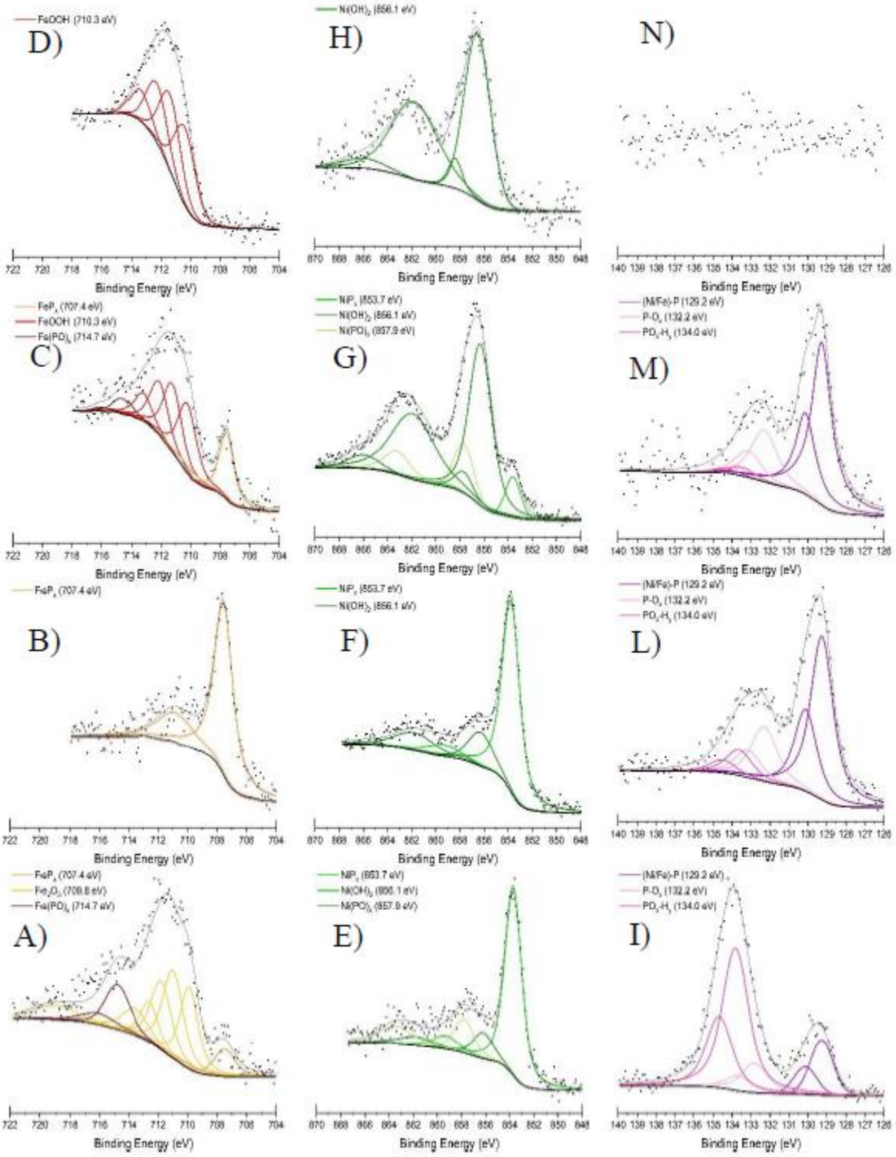

Figure S7. XPS data of $Fe_{0.25}Ni_{0.75}P_x$ on CC: (**A**) Fe 2p as-prepared, (**B**) Fe 2p post-HER in acid solution, (**C**) Fe 2p post-HER in alkaline solution, (**D**) Fe 2p post-OER in alkaline solution, (**E**) Ni 2p as-prepared, (**F**) Ni 2p post-HER in acid solution, (**G**) Ni 2p post-HER in alkaline solution, (**H**) Ni 2p post-OER in alkaline solution, (**I**) P 2p as-prepared, (**L**) P 2p post-HER in acid solution, (**M**) P 2p post -HER in alkaline solution, (**N**) P 2p post-OER in alkaline solution.

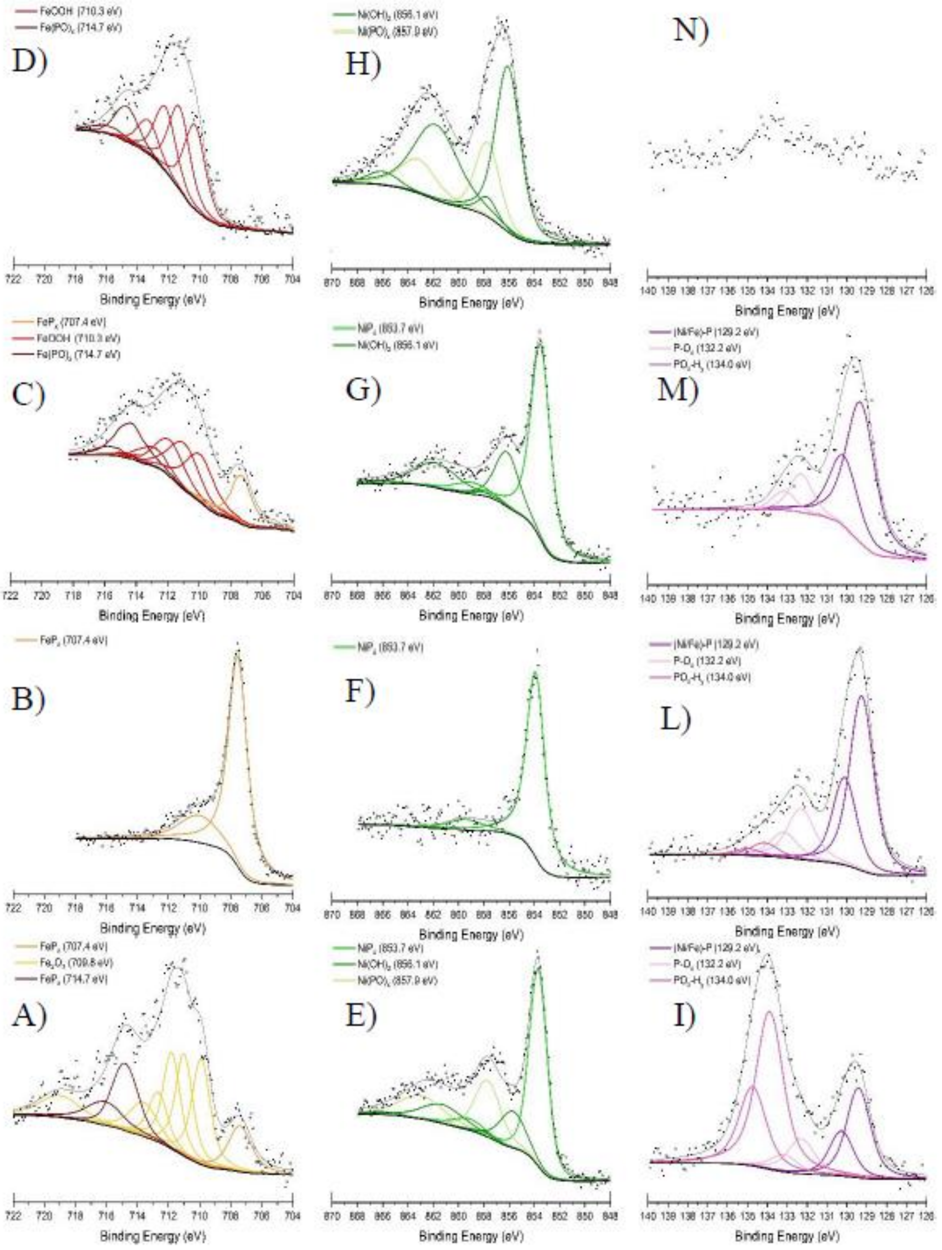

Figure S8. XPS data of $Fe_{0.75}Ni_{0.25}P_x$ on CC: (**A**) Fe 2p as-prepared, (**B**) Fe 2p post-HER in acid solution, (**C**) Fe 2p post-HER in alkaline solution, (**D**) Fe 2p post-OER in alkaline solution, (**E**) Ni 2p as-prepared, (**F**) Ni 2p post-HER in acid solution, (**G**) Ni 2p post-HER in alkaline solution, (**H**) Ni 2p post-OER in alkaline solution, (**I**) P 2p as-prepared, (**L**) P 2p post-HER in acid solution, (**M**) P 2p post -HER in alkaline solution, (**N**) P 2p post-OER in alkaline solution.

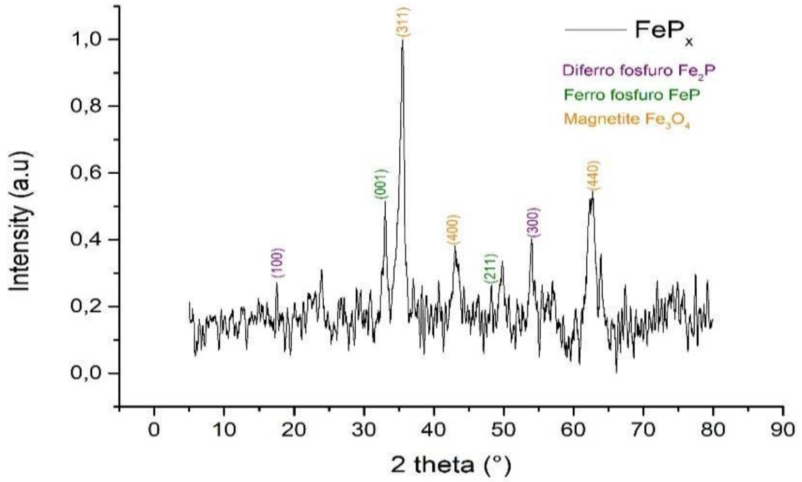

Figure S9. XRD pattern of FeP$_x$. Peaks related to Fe_2P, FeP and Fe_3O_4 are labelled in purple, green and yellow, respectively.

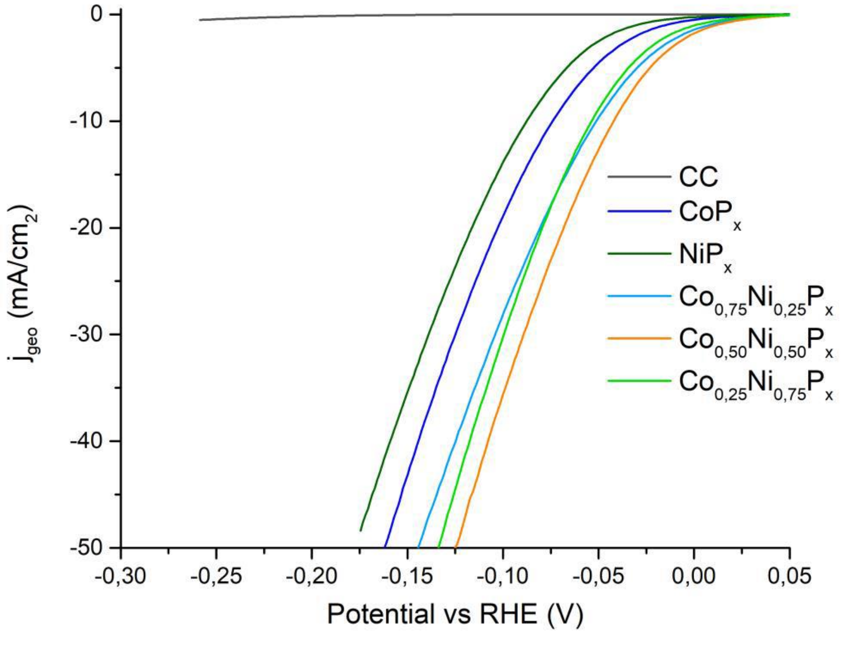

Figure S10. HER EC in acid electrolyte for CC with Co and Ni phosphides.

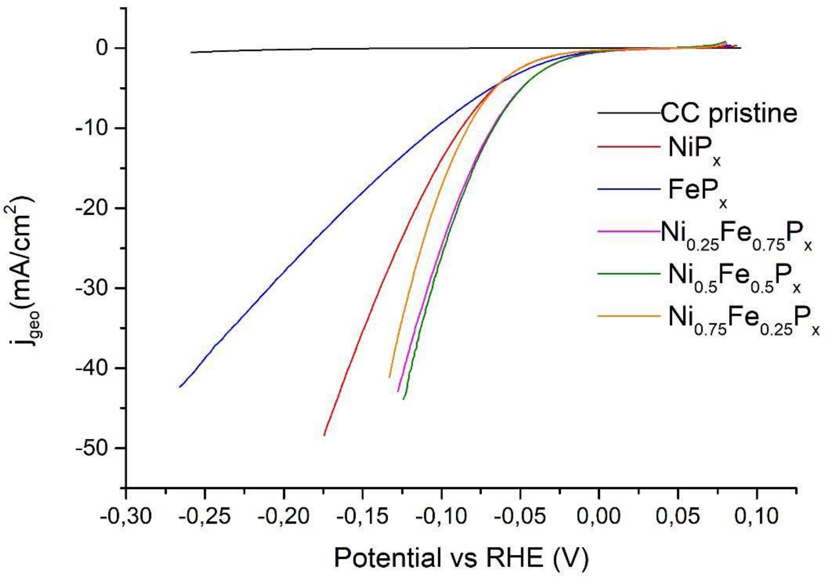

Figure S11. HER EC in acid electrolyte for CC with Fe and Ni phosphides.

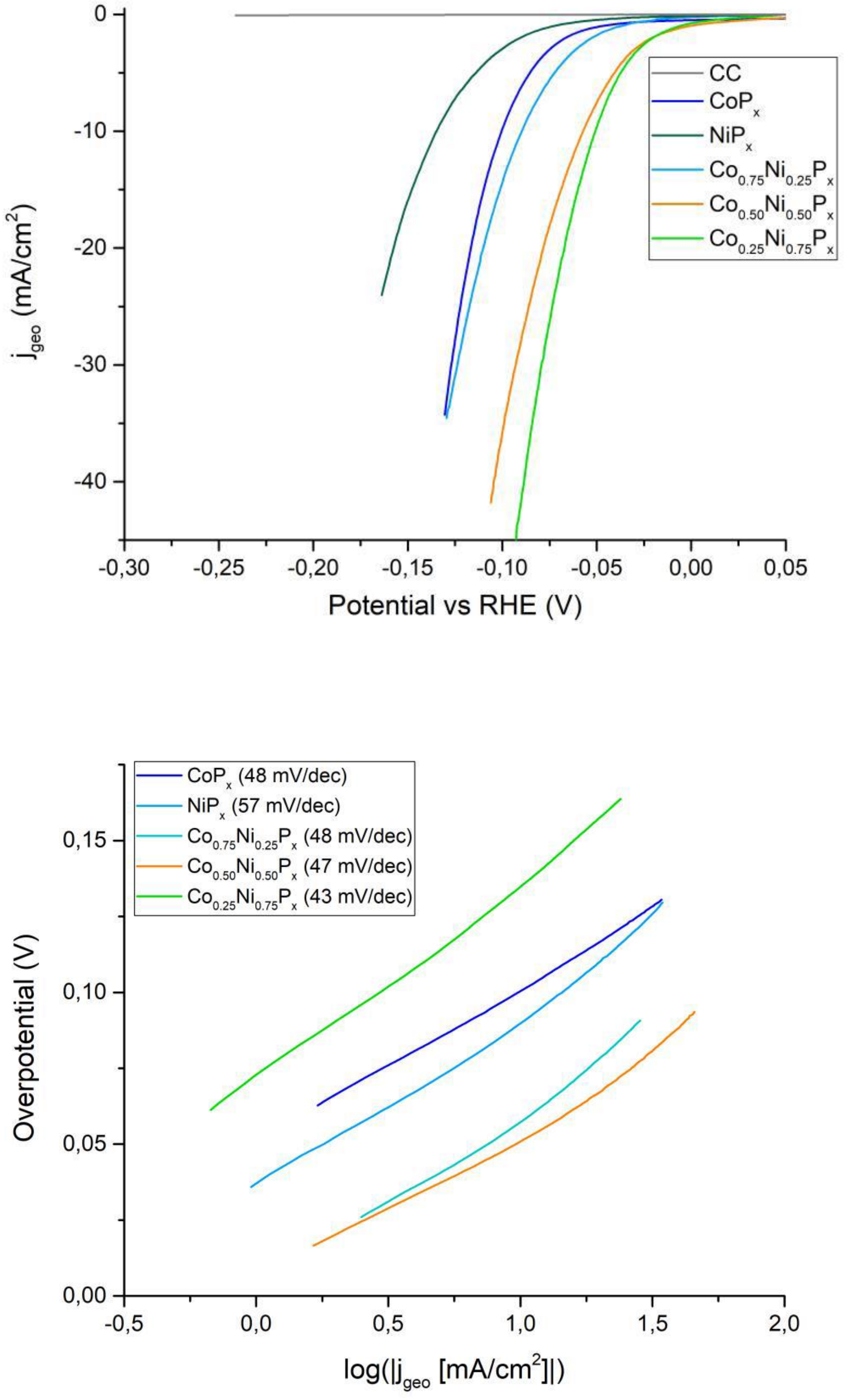

Figure S12. HER EC in alkaline electrolyte for CC with Co and Ni phosphides.

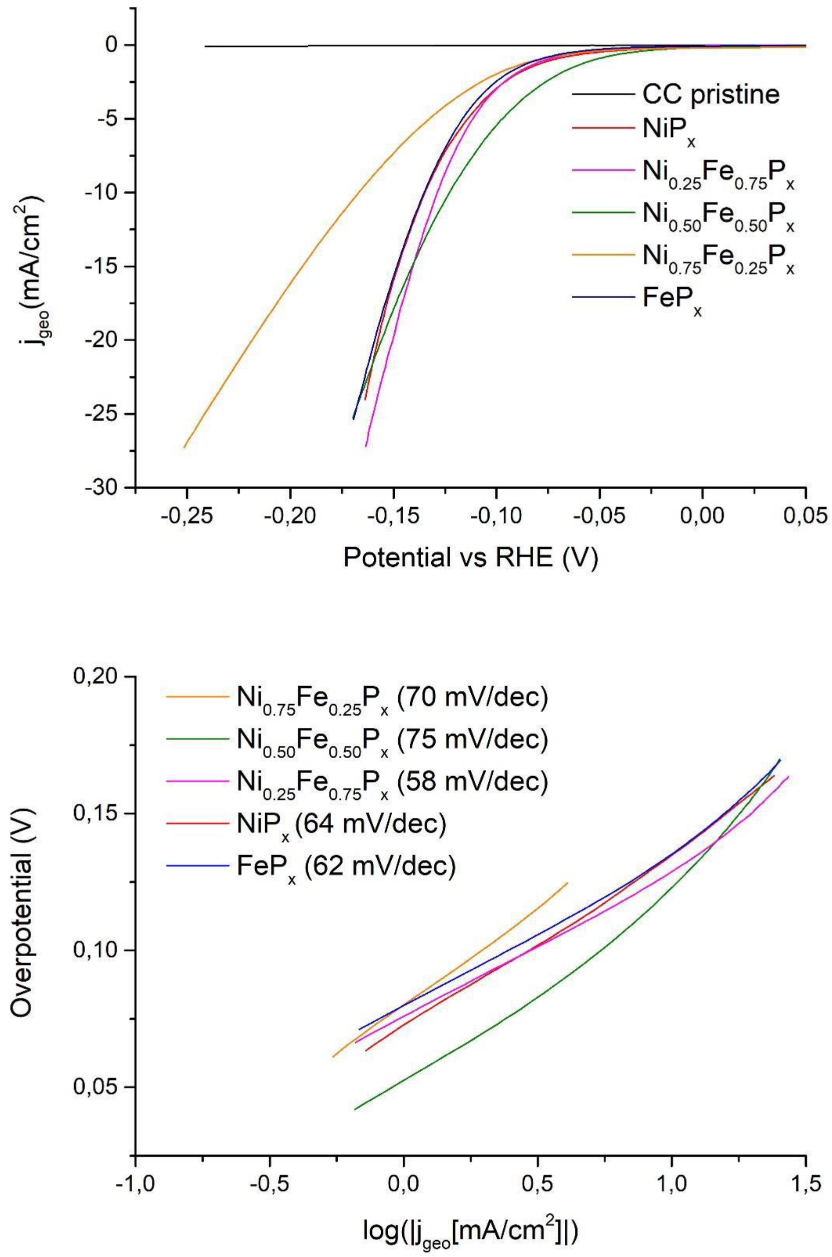

Figure S13. HER EC in alkaline electrolyte for CC with Fe and Ni phosphides.

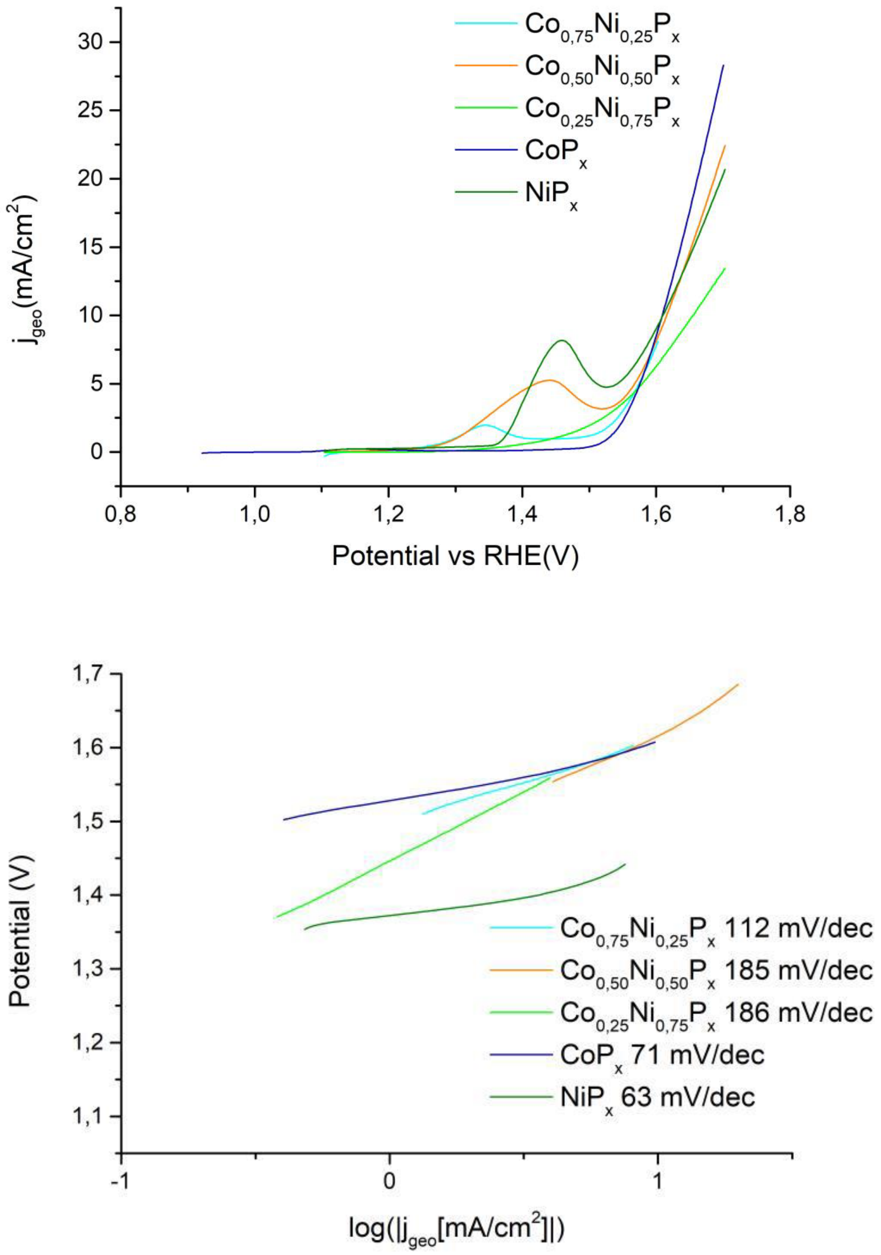

Figure S14. OER EC in alkaline electrolyte for CC with Co and Ni phosphides.

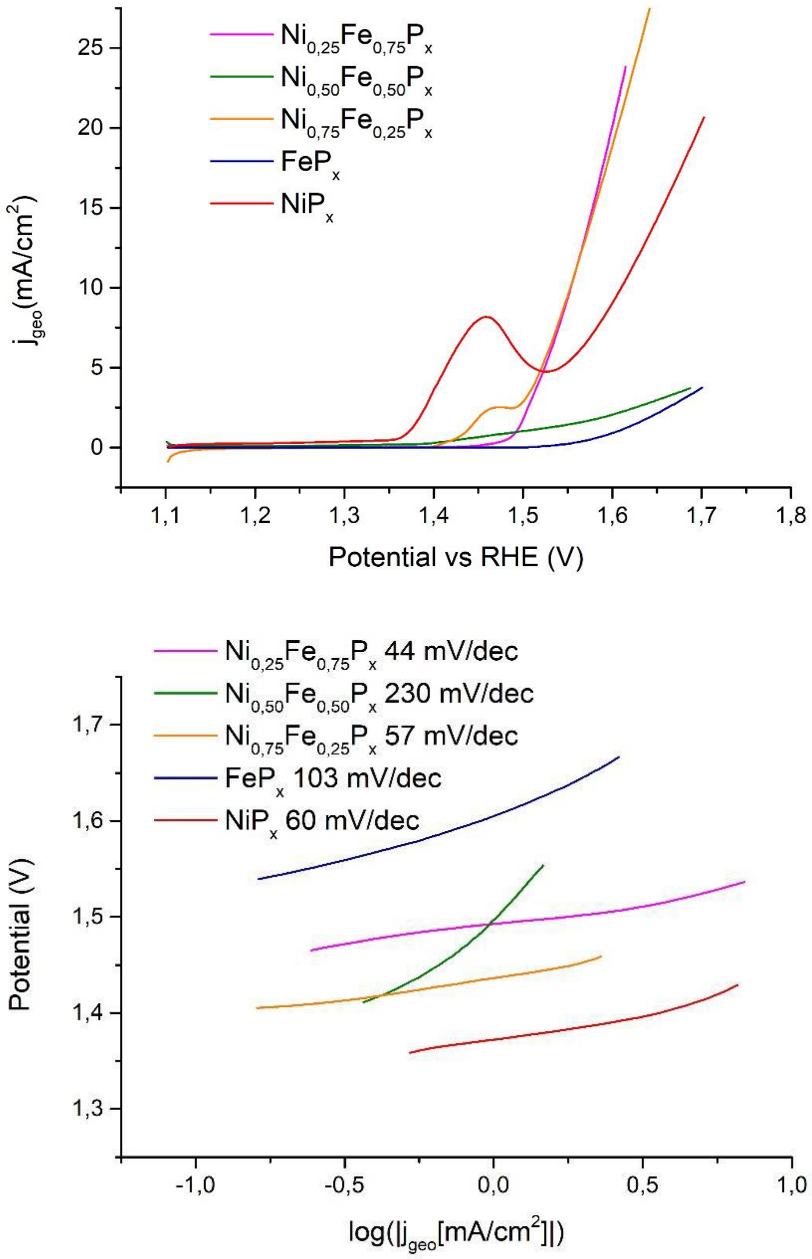

Figure S15. OER EC in alkaline electrolyte for CC with Fe and Ni phosphides.
